# Anatomical variations of the superior labial branch of the facial artery

**DOI:** 10.1016/j.jpra.2026.02.028

**Published:** 2026-03-01

**Authors:** Mamatha Hosapatna, Jyotsna Bailur, Aamna Kausar, Veeresh Hosmani, Veeresh

**Affiliations:** a Department of Anatomy, Kasturba Medical College, Manipal Academy of Higher Education (MAHE), Manipal, Karnataka, India; b Division of Anatomy, Department of Basic Medical Sciences (DBMS), Manipal Academy of Higher Education (MAHE), Manipal, Karnataka, India; c Department of Anatomy, Koppal Institute of Medical Sciences (KIMS), Koppal, Karnataka, India

**Keywords:** Superior labial artery, Inferior labial artery, Vascular anatomy, Cheilion, Gonion

## Abstract

A precise understanding of the facial artery and its labial branches is crucial for safe surgical planning in the perioral and nasal regions. This study aimed to describe the branching patterns of the superior labial artery and to establish morphometric relationships of the facial artery, superior labial artery, and inferior labial artery with surface landmarks in the Indian population. Thirty hemifacial specimens from formalin-fixed adult cadavers were dissected. The origin and branching configurations of the superior labial artery and inferior labial artery were recorded, and horizontal and vertical distances from the cheilion and gonion to the Facial artery, superior labial artery, and inferior labial artery were measured using a digital vernier calliper (0.01-mm accuracy).

Four superior labial artery distribution patterns were identified, with Type 1 being the most prevalent (70 %). The mean gonion-to-facial artery distance was 2.20 ± 0.25 cm on the left and 2.20 ± 0.44 cm on the right. The cheilion-to-facial artery distance measured 1.45 ± 0.26 cm (left) and 1.44 ± 0.33 cm (right). The superior labial artery originated superior to the cheilion, whereas the inferior labial artery originated inferior to it. Superior labial artery vertical distances were 0.76 ± 0.39 cm (left) and 0.70 ± 0.41 cm (right), while inferior labial artery distances measured 1.75 ± 0.42 cm (left) and 2.00 ± 0.46 cm (right).

These findings reveal notable variation in the anatomy of the superior labial artery and provide consistent surface-based morphometric references that are helpful for safer facial reconstructive and aesthetic procedures.

## Introduction

A precise understanding of vascular anatomy is indispensable for surgical planning and radiologic interpretation. Owing to its aesthetic and functional significance, the face is frequently affected by trauma, making detailed anatomical knowledge crucial for accurate diagnosis and intervention.^1^ The increasing demand for facial surgical and reconstructive procedures further underscores the importance of defining reliable vascular patterns.^2^ The facial artery (FA) constitutes the major arterial supply to the face, providing perfusion to the upper and lower lips via the superior labial artery (SLA) and inferior labial artery (ILA). The dense vascular network of the facial region contributes to rapid tissue healing and favorable surgical outcomes.^3^

The FA originates as an anterior branch of the external carotid artery (ECA). It ascends over the submandibular gland, crosses the inferior margin of the mandible near the anterior border of the masseter muscle, and proceeds along a tortuous course toward the oral commissure. It then continues alongside the nasal border, terminating near the medial canthus of the eye.^4^

Along its route on the face, the FA gives rise to several branches, including the SLA, ILA, and the Lateral nasal artery (LNA), which supply the upper lip, lower lip, and dorsum of the nose, respectively. The artery ultimately terminates as the angular artery, which supplies the medial canthus of the eye.^5^

Variations in FA morphology, such as agenesis, hypoplasia, and enlargement, have been documented and hold significant clinical relevance.^6^ Most previous studies have focused on describing FA branching patterns and their prevalence, as this knowledge is fundamental for performing aesthetic and reconstructive facial procedures safely while minimizing complications such as hemorrhage and tissue necrosis.^7^ However, there remains a need for population-specific anatomical data, particularly in the Karavali region of South India, to guide clinicians in planning safe and effective interventions.

The present study, therefore, investigated the distribution pattern of the SLA and examined the morphometric relationships of the FA and its branches with reliable surface landmarks, providing clinically applicable data for improved intraoperative localization and surgical outcomes.

## Materials and methods

**Figure 1:**
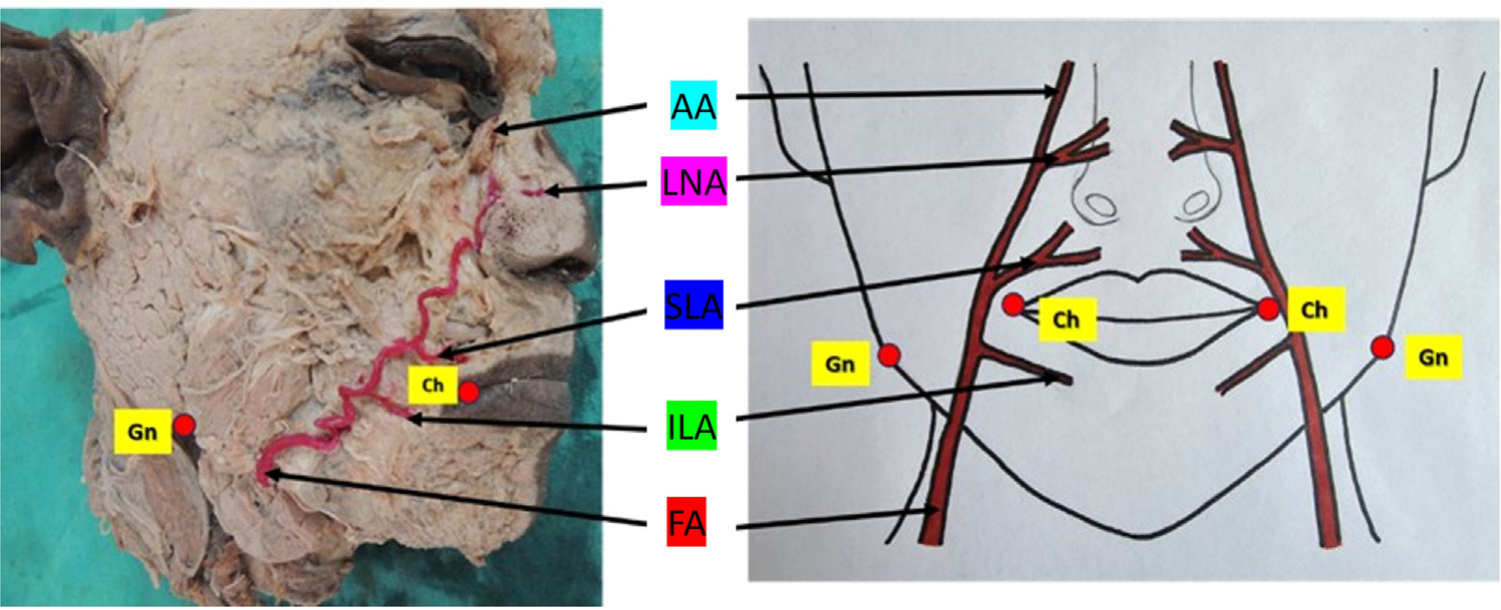
Illustration of the branches of the FA, including a schematic diagram depicting its various branches.

The study was conducted on 30 hemifacial specimens obtained from formalin-fixed adult cadavers of unknown sex, comprising 15 right and 15 left hemifaces, sourced from the Department of Anatomy. Before initiation, ethical approval was obtained from the Institutional Ethics Committee [IEC-344 /2025] (Figure 1)

**Figure 2:**
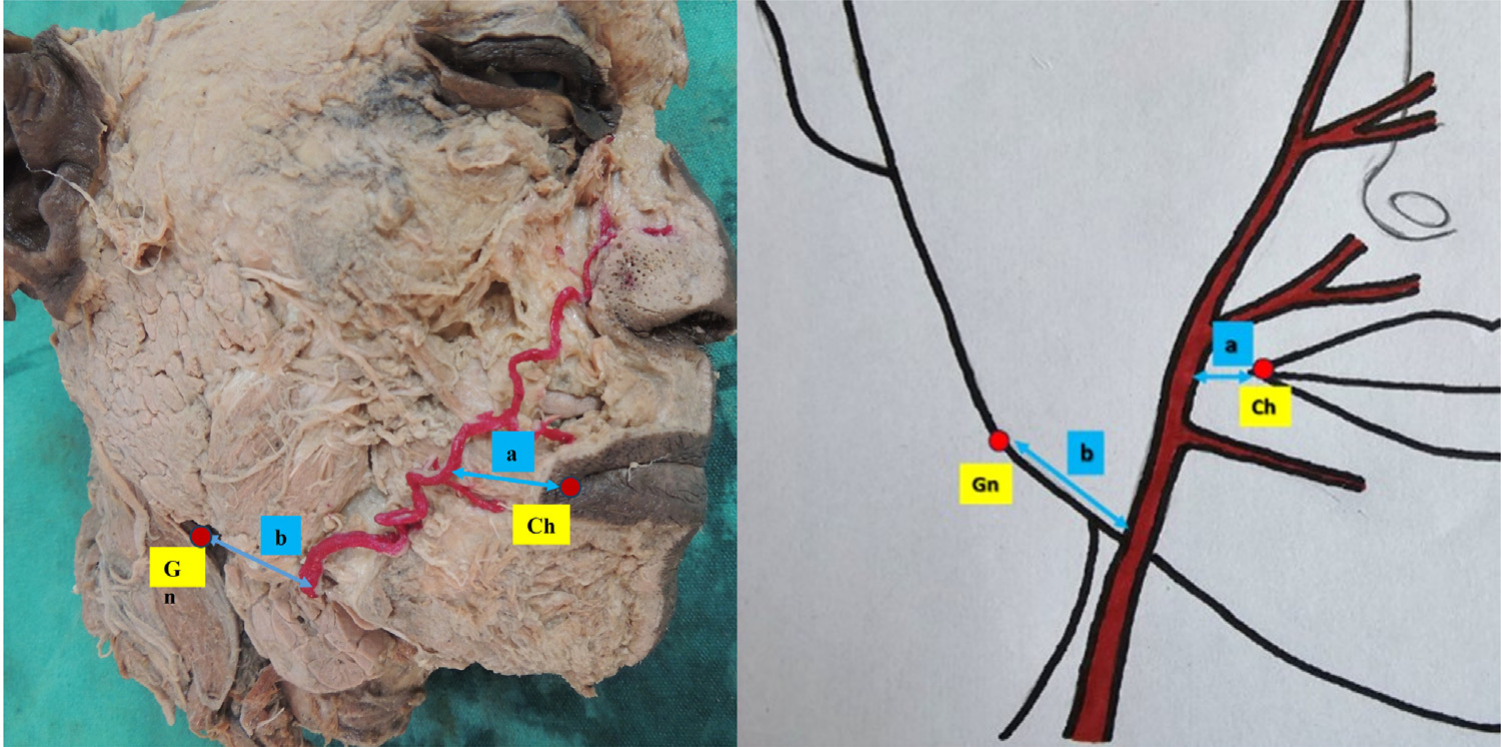
Illustration showing the measurement parameters: *a* – Horizontal distance of the FA from the Ch; *b* – Distance between the FA and the Gn along the inferior border of the mandible.

Topographical measurements of the FA and the origins of the SLA and ILA were obtained using the cheilion (Ch) and gonion (Gn) as fixed anatomical landmarks. The parameters recorded included:

(a) The horizontal distance of the FA from the Ch; and
(b) The distance between the FA and the Gn along the inferior border of the mandible.as shown in Figure 2.

**Figure 3:**
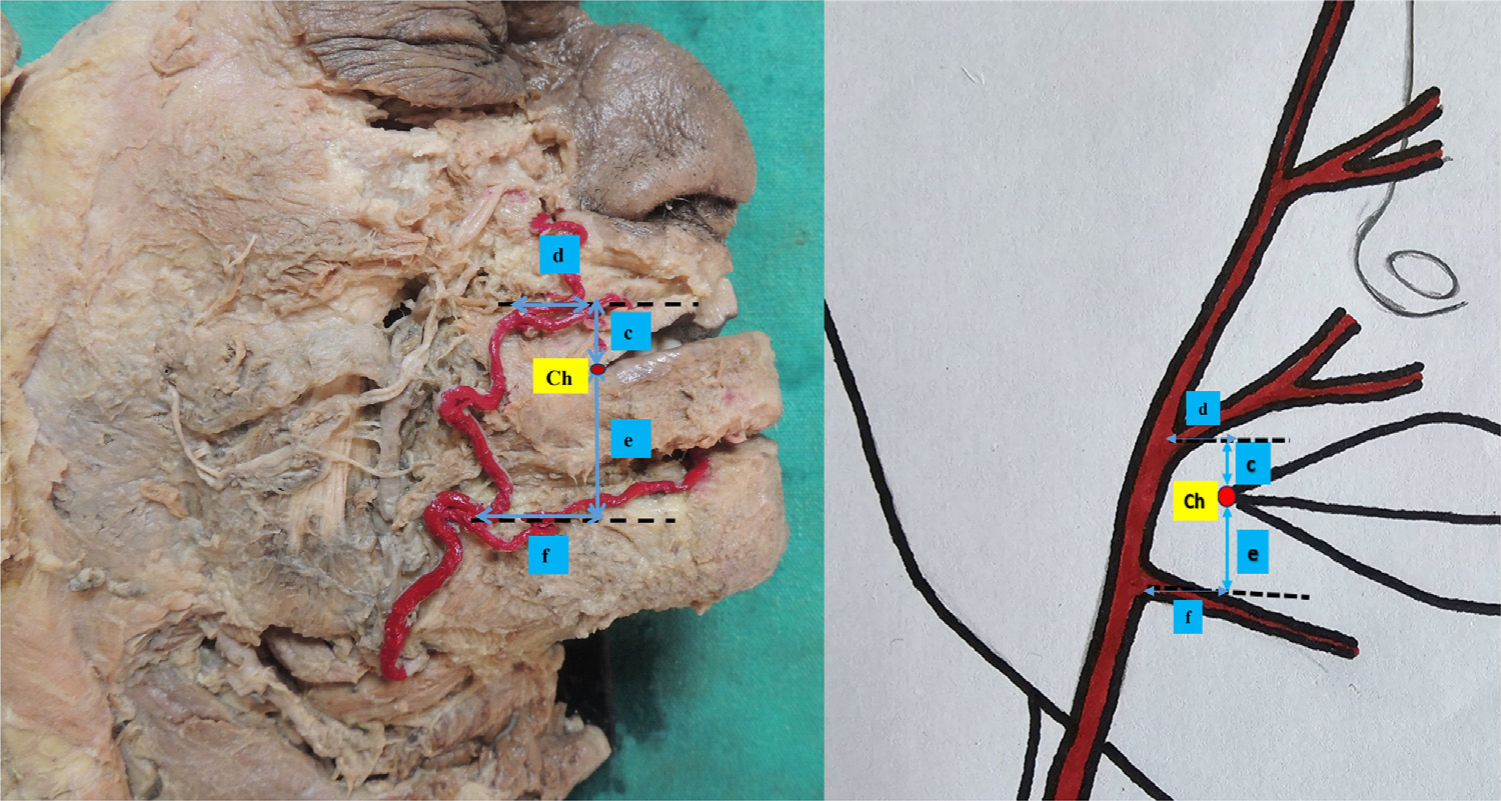
Illustration showing the parameters used for measurement. c – Vertical distance of the SLA from the Ch. d– Horizontal distance of the SLA from the Ch. e – Vertical distance of the ILA from the Ch. f – Horizontal distance of the ILA from the Ch.

For each arterial origin, a horizontal reference line was drawn through the branching point, and the vertical distance from the Ch to this line was measured. Similarly, a vertical reference line was drawn through the Ch, and the horizontal distance from the arterial origin to this line was recorded. All measurements were taken using a digital vernier caliper with an accuracy of 0.01 mm. The remaining parameters included:

(c) The vertical distance of the SLA from the Ch;
(d) The horizontal distance of the SLA from the Ch;
(e) The vertical distance of the ILA from the Ch; and
(f) The horizontal distance of the ILA from the Ch. As shown in Figure 3.

## Results

### Morphological results

**Figure 4:**
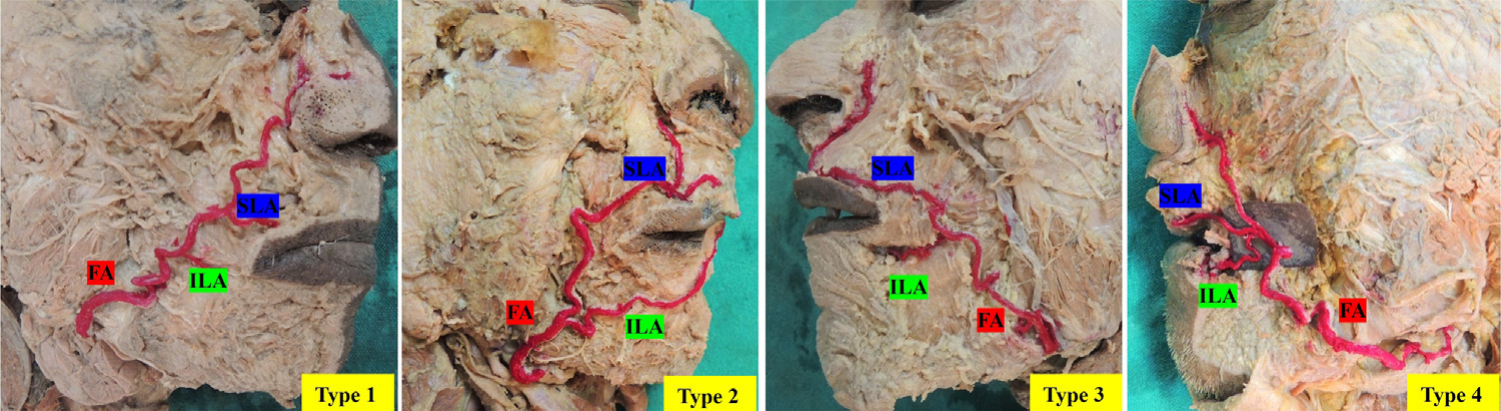
Showing the Various distribution patterns of SLA and ILA. Type 1: FA gives ILA and SLA branches and continues upward. Type 2: FA gives ILA branch and continues as SLA; SLA provides a branch that runs upward on the lateral aspect of the alae of the nose. Type 3: FA gives an ILA branch and continues as SLA, which runs towards the lateral aspect of the alae from the columella. Type 4: SLA and ILA arise from the common trunk of the FA.

The study identified distinct patterns of SLA distribution, consistent with those described in the literature. The Type 1 pattern was the most common, observed in 70 % of specimens, followed by Type 3 (20 %), Type 2 (6.7 %), and Type 4 (3.3 %). Overall, four SLA distribution patterns were observed, as shown in Figure 4.

### Morphometric results

The morphometric parameters of the FA and its labial branches were measured bilaterally with reference to the Ch and Gn. The mean values and standard deviations of all measurements are presented in Table 1. The mean distance between the Gn and FA was 2.20 ± 0.25 cm on the left side and 2.20 ± 0.44 cm on the right side. The mean horizontal distance between the Ch and FA measured 1.45 ± 0.26 cm on the left and 1.44 ± 0.33 cm on the right. With respect to the SLA, the mean vertical distance from the Ch to the origin of the SLA was 0.76 ± 0.39 cm on the left and 0.70 ± 0.41 cm on the right. In comparison, the corresponding horizontal distances were 1.00 ± 0.61 cm and 0.90 ± 0.82 cm, respectively. For the ILA, the mean vertical distance from the Ch was 1.75 ± 0.42 cm on the left and 2.00 ± 0.46 cm on the right. The mean horizontal distance from the Ch to the origin of the ILA measured 1.73 ± 0.55 cm on the left and 1.90 ± 0.51 cm on the right. Left- and right-side measurements were compared descriptively. Inferential statistical analysis was not performed due to the limited sample size and the use of hemifacial specimens.

**Table 1:** Morphometric measurements of the FA and its labial branches in relation to the Ch and Gn. (all measurements in cm).

Side	Gn to FA	Ch to FA	Ch to the origin of SLA		Ch to the origin of ILA	
			Vertical	Horizontal	Vertical	Horizontal
Left	2.2 ± 0.25	1.45 ± 0.26	0.76 ± 0.39	1.0 ± 0.61	1.75 ± 0.42	1.73 ± 0.55
Right	2.2 ± 0.44	1.44 ± 0.33	0.7 ± 0.41	0.9 ± 0.82	2.0 ± 0.46	1.9 ± 0.51

## Discussion

Precise anatomical knowledge of the variations and surface relationships of the facial artery and its labial branch is essential for surgical and non-surgical facial aesthetic procedures, as successful planning and execution depend heavily on an accurate understanding of the region’s vascular anatomy.^8^ Variability in the morphometry and branching patterns of these vessels directly affects dermal filler and neurotoxin injections in the perioral region, where unrecognized arterial proximity may result in serious complications, including tissue necrosis, visual impairment, stroke, or death.^9^^,^^10^ Precise morphometric assessment using consistent surface landmarks, such as the cheilion and gonion, allows clinicians to estimate arterial location, identify potential danger zones, and optimize injection accuracy.^11^^,^^12^ Recognition of individual anatomical differences supports customized treatment planning rather than standardized approaches.

Anatomical variability of the FA and its branches has been extensively documented. Loukas et al. examined variations in FA, with particular emphasis on the SLA, and identified five distinct branching types. In Type A, the most common pattern (47.5 %), the FA bifurcates into the SLA and the LNA. Type B (38.7 %) is characterized by the LNA terminating as the superior alar artery, with no identifiable angular artery. In Type C (8.4 %), the FA itself terminates as the SLA. Type D (3.8 %) describes a configuration in which the angular artery arises directly from the FA trunk rather than as a continuation of the LNA, and the FA subsequently terminates as the superior alar artery. The least common variant, Type E (1.4 %), involves the FA ending as a rudimentary twig lacking significant terminal branches.^13^

Nakajima et al. described three principal distribution patterns of the FA. In Type A, the FA bifurcates into the SLA and the LNA. Type B is characterized by the LNA terminating as the superior alar artery. In Type C, the FA itself terminates as the SLA.^14^ These findings were supported by Crouzet et al.^15^ While Schulte et al. Observed, in 15 of 16 cadavers, an FA that bifurcated into the SLA and the angular artery.^16^

Al-Hoqail RA et al. mentioned that the ILA formed a common trunk with the SLA in 28.6 %.^17^

Angulo CK et al. reported five principal patterns describing the topographic and morphological distribution of the FA. In 47.5 % of cases, the FA bifurcates into the SLA and the LNA, with the LNA continuing as the angular artery. In 38.7 %, the FA terminates as the superior alar artery. In 8.4 %, the FA ends as the SLA. In 3.8 %, an angular branch arises below the oral commissure directly from the FA trunk, and the FA subsequently terminates as the superior alar artery. The least common pattern, occurring in 1.4 % of cases, involves the FA terminating as a rudimentary branch lacking significant SLA, LNA, or angular arteries.^2^

Lee Sang-Hee et al. further characterized the SLA by identifying four distinct patterns of origin. In Type I, the most common configuration (56.7 %), the SLA and the alar branch arise directly and independently from the FA. Type II (21.7 %) features the SLA branching from the FA and subsequently giving rise to an alar branch. In Type III (15.0 %), the SLA represents the terminal branch of the FA. Type IV, the least frequent pattern (6.7 %), is defined by the absence of the SLA.^18^

K Niemann et al. reported considerable variability in the termination of the FA. According to their findings, the FA may terminate as the ILA (5.13 %), the SLA (10.26 %), the inferior alar artery (10.26 %), the superior alar artery (46.15 %), the LNA (5.13 %), or the angular artery (20.51 %).^19^

In the present study, four distinct branching patterns of the FA were observed. In Type 1, the FA gives rise to both the ILA and the SLA before continuing its course superiorly. Type 2 consists of the FA giving off the ILA and then continuing as the SLA, with the SLA issuing an additional branch that ascends along the lateral aspect of the alae of the nose. In Type 3, the FA provides the ILA and continues as the SLA, which then courses toward the lateral aspect of the alae from the region of the columella. Type 4 demonstrates a configuration in which the SLA and ILA arise together from a common trunk originating from the FA.

The morphometric findings of the present study show several differences when compared with previously published measurements. The distance from the Gn to the FA in our specimens was 2.2 ± 0.25 cm on the left and 2.2 ± 0.44 cm on the right, which is notably shorter than the values reported in earlier studies, where the mean distance measured 3.34 ± 0.36 cm on the right and 3.23 ± 0.34 cm on the left.^11^ The distance between the Ch and the FA in our material measured 1.45 ± 0.26 cm on the left and 1.44 ± 0.33 cm on the right, which falls within the broad range of previously published values, including 15.3 ± 3.7 mm.^20^ 19 ± 5.5 mm in cadavers and 18 ± 4.0 mm in living subjects,^21^ 8.5 ± 4.0 mm.^9^

The origin of the SLA was located superior to the Ch in all cases, with vertical distances of 0.76 ± 0.39 cm (left) and 0.70 ± 0.41 cm (right), and horizontal distances of 1.0 ± 0.61 cm (left) and 0.9 ± 0.82 cm (right). These findings agree with earlier reports that the SLA origin lies above the mouth corner in 68.51 % to 91.01 % of cases.^10^^,^^22^ And at an average distance of approximately 1.29 cm from the commissure.^10^^,^^23^ Our measured horizontal distance is also comparable to the reported mean of 12.1 ± 3.1 mm lateral to the Ch.^18^

The ILA originated at vertical distances of 1.75 ± 0.42 cm (left) and 2.0 ± 0.46 cm (right), and at horizontal distances of 1.73 ± 0.55 cm (left) and 1.9 ± 0.51 cm (right) from the Ch. These findings correspond well with previous descriptions, placing the ILA origin approximately 23.9 mm from the labial commissure.^24^

Together, these morphometric observations confirm considerable variability in the course and origin of the FA and its branches, while remaining consistent with the established ranges reported in the literature.

Detailed knowledge of the course and variable positions of the SLA is essential to avoid complications, such as producing a non-viable Abbe flap. This can occur due to inadvertent ligation of the labial artery or disruption of its critical anastomoses.^25^ For more invasive procedures like facelifts or lip reconstructions, understanding the facial artery's branching patterns and termination points is crucial. Variations in arterial anatomy can affect surgical planning and outcomes.^26^

## Conclusion

The present study highlights significant variations in the origin and branching pattern of the SLA in the Karavali region of South India, with Type 1 emerging as the most frequent configuration (70 %), characterized by the FA giving rise to both the ILA and SLA before ascending. The morphometric analysis demonstrated consistent, clinically valuable relationships among the FA, SLA, ILA, and nearby surface landmarks, reinforcing their importance for precise surgical localization. Overall, these findings emphasize the need for comprehensive anatomical knowledge of FA variations to optimize surgical planning, minimize the risk of intraoperative vascular injury, and enhance outcomes in facial reconstructive and aesthetic procedures.

However, the use of hemifacial specimens of unknown sex and age, limited sample size, and the inability to reliably assess arterial depth due to variable soft-tissue thickness in embalmed cadavers represent limitations.

These limitations should be considered when interpreting the findings in a clinical context, alongside existing anatomical studies.

## Funding

None.

## Declaration of competing interest

The authors have no relevant financial or non-financial interests to disclose.
